# The Characterization of *Escherichia coli* CpdB as a Recombinant Protein Reveals that, besides Having the Expected 3´-Nucleotidase and 2´,3´-Cyclic Mononucleotide Phosphodiesterase Activities, It Is Also Active as Cyclic Dinucleotide Phosphodiesterase

**DOI:** 10.1371/journal.pone.0157308

**Published:** 2016-06-13

**Authors:** Iralis López-Villamizar, Alicia Cabezas, Rosa María Pinto, José Canales, João Meireles Ribeiro, José Carlos Cameselle, María Jesús Costas

**Affiliations:** Grupo de Enzimología, Departamento de Bioquímica y Biología Molecular y Genética, Facultad de Medicina, Universidad de Extremadura, Badajoz, Spain; University of Kentucky College of Medicine, UNITED STATES

## Abstract

Endogenous cyclic diadenylate phosphodiesterase activity was accidentally detected in lysates of *Escherichia coli* BL21. Since this kind of activity is uncommon in Gram-negative bacteria, its identification was undertaken. After partial purification and analysis by denaturing gel electrophoresis, renatured activity correlated with a protein identified by fingerprinting as CpdB (*cpdB* gene product), which is annotated as 3´-nucleotidase / 2´,3´-cyclic-mononucleotide phosphodiesterase, and it is synthesized as a precursor protein with a signal sequence removable upon export to the periplasm. It has never been studied as a recombinant protein. The coding sequence of mature CpdB was cloned and expressed as a GST fusion protein. The study of the purified recombinant protein, separated from GST, confirmed CpdB annotation. The assay of catalytic efficiencies (*k*_cat_/*K*_m_) for a large substrate set revealed novel CpdB features, including very high efficiencies for 3´-AMP and 2´,3´-cyclic mononucleotides, and previously unknown activities on cyclic and linear dinucleotides. The catalytic efficiencies of the latter activities, though low in relative terms when compared to the major ones, are far from negligible. Actually, they are perfectly comparable to those of the ‘average’ enzyme and the known, bona fide cyclic dinucleotide phosphodiesterases. On the other hand, CpdB differs from these enzymes in its extracytoplasmic location and in the absence of EAL, HD and DHH domains. Instead, it contains the domains of the 5´-nucleotidase family pertaining to the metallophosphoesterase superfamily, although CpdB lacks 5´-nucleotidase activity. The possibility that the extracytoplasmic activity of CpdB on cyclic dinucleotides could have physiological meaning is discussed.

## Introduction

The *cpdB* gene is widespread among prokaryotes, although most studies are focused on Gram-negative bacteria. The *E*. *coli cpdB* gene was identified and cloned by Beacham and coworkers [1] who found that it encodes the precursor of a periplasmic protein. *E*. *coli cpdB* mutants are devoid of the 3´-nucleotidase and 2´,3´-cyclic-nucleotide phosphodiesterase activities previously studied in bacterial extracts [2, 3]. This defect is complemented by transformation with DNA fragments containing the *cpdB* gene [1]. The homologous gene of *Yersinia enterocolitica* has also been cloned and *cpdB* mutants are unable to grow on 2´,3´-cAMP as the only carbon source, defect also complemented by transformation with *cpdB* [4]. Very recently, the role of *cpdB* in the growth of *Vibrio cholerae* supported by extracellular DNA as source of carbon and phosphorus has been demonstrated [5]. Also recently, the *cpdB* gene of *Salmonella enterica* has been shown to increase the intracellular persistence of the pathogen in infected chicken [6]. Despite the availability of this information on *cpdB*, the annotation of the genes and their protein products (CpdB) as 3´-nucleotidase / 2´,3´-cyclic-nucleotide phosphodiesterase relies just on the absence of these activities in *cpdB* mutant bacteria, or just on sequential homology. All the previous specificity and kinetic studies of 3´-nucleotidase / 2´,3´-cyclic-nucleotide phosphodiesterase have been run with enzyme obtained by purification from bacterial extracts [2, 3, 5, 7–13]. Although the *cpdB* gene has been studied previously, to our knowledge, the CpdB protein has not been expressed and characterized enzymatically as a recombinant protein.

Bacterial cyclic dinucleotides are regulators that affect multiple aspects of prokaryotic physiology, pathogenicity, and interaction with the infected host and its immune system. Best known of these compounds is 3´,5´-cyclic diguanylate (c-di-GMP). It was discovered as a regulator of cellulose synthesis by *Gluconoacetobacter* [14], and currently constitutes a major topic in bacterial research. C-di-GMP controls, among other things, motility, biofilm formation, virulence and cell cycle progression of Gram-negative bacteria (reviewed in [15–19]), and their interaction with the innate immune system of the infected host [20]. The c-di-GMP analog 3´,5´-cyclic diadenylate (c-di-AMP) was discovered more recently within a protein crystal of *Bacillus subtilis* DisA (DNA integrity scanning protein A), which synthesizes the dinucleotide [21]. In this system, c-di-AMP couples DNA integrity with progression of sporulation [22] and with stress homeostasis during vegetative growth [23]. However, further research is making evident that it also has multiple additional effects in Gram-positive bacteria (reviewed in [19, 24, 25]), including cell wall homeostasis [26, 27], allosteric regulation of metabolic enzyme function [28], mediation of biofilm formation [29] and induction of type I interferon (IFN) response in the infected host [30]. There is limited but significant evidence that c-di-AMP is produced by (some) Gram-negative organisms. This has been noted in *Chlamydia trachomatis* [31] and *Borrelia burgdorferi* [32, 33].

This work was started because, while pursuing the cloning and expression of a possible c-di-AMP phosphodiesterase from Gram-positive bacteria (the *Peptoclostridium difficile* homolog of GdpP [34]), we found accidentally that the *E*. *coli* BL21 cells to be used as the expression host contained endogenous c-di-AMP phosphodiesterase activity. By then this kind of enzyme activity had not been described in Gram-negative cells, although more recently it has been shown that *B*. *burgdorferi* DhhP hydrolyzes c-di-AMP [32]. This is still a rather isolated observation, and there is no report of c-di-AMP hydrolysis in proteobacteria. Therefore, the molecular identification of the *E*. *coli* enzyme active as c-di-AMP phosphodiesterase was undertaken.

Here we are reporting the identification of CpdB as the endogenous c-di-AMP phosphodiesterase of *E*. *coli*, and its first characterization as a recombinant protein, with a detailed study of substrate specificity and kinetics. The results give direct biochemical (i.e. protein-based) support for the current annotation of *cpdB* gene and protein as 3´-nucleotidase / 2´,3´-cyclic-mononucleotide phosphodiesterase. In addition, the assay of catalytic efficiencies (*k*_cat_/*K*_m_) revealed novel CpdB features, including its very high efficiency as 3´-nucleotidase / 2´,3´-cyclic-mononucleotide phosphodiesterase, and previously unknown activities on cyclic and linear dinucleotides.

## Materials and Methods

### Bacterial strains

*E*. *coli* JM109 competent cells (Promega) were used for cloning and subcloning procedures. *E*. *coli* BL21 Gold competent cells (Agilent Technologies) were used as the source of endogenous cyclic diadenylate phosphodiesterase, or of genomic DNA for PCR amplification of the coding sequence of mature CpdB, and for recombinant protein expression. The commercial strains were stored at -80°C.

### Purification of a protein band associated with cyclic diadenylate phosphodiesterase activity from soluble lysates of *E*. *coli* BL21 cells

Fifteen microliters of thawed BL21 cells were suspended in 1 ml and inoculated in 200 ml of LB medium (10 g l^-1^ bacto-tryptone, 5 g l^-1^ yeast extract, 10 g l^-1^ NaCl). After 14 h at 37°C with 220 rpm shaking (when *A*_600_ reached a value of 1.5), cells were collected by centrifugation for 15 min at 4000 × g and 4°C. The cell pellet was resuspended in 10 ml of 20 mM Tris-HCl, pH 7.5 at 4°C, 0.5 mM EDTA, 1 mM dithiothreitol, 50 mM NaCl. Cells kept on ice were lysed by sonication with three 5-min series of 0.1 s pulses per second at 170 W, and 5-min waiting between series. The lysate was supplemented with 5 g l^-1^ Triton X-100 and, after 30 min of mild agitation at 4°C, it was centrifuged for 30 min at 85000 × g and 4°C to collect 11 ml of supernatant (soluble lysate).

Four milliliters of soluble lysate was chromatographed in a Sephadex G-100 column (90 cm × 1 cm) pre-equilibrated and eluted at 0.25 ml/min with 20 mM Tris-HCl, pH 7.5 at 4°C, 0.5 mM EDTA, 100 mM NaCl, 2 g l^-1^ Triton X-100. Fractions of ≈ 2 ml were collected, and protein content [35] and phosphodiesterase activities on c-di-AMP and bis-4-nitrophenylphosphate were assayed (see below). A 10-ml pool of seven fractions of a peak of cyclic diadenylate phosphodiesterase activity eluting at V_e_ ≈ 60 ml was dialyzed twice against 100 volumes of elution buffer devoid of NaCl.

The dialysate of the previous step was applied to a Q-Sepharose column (11 cm × 1 cm) pre-equilibrated with 20 mM Tris-HCl, pH 7.5 at 4°C. Sample application was followed by a 12-ml wash with 20 mM Tris-HCl, pH 7.5 at 4°C, 0.5 mM EDTA, 2 g l^-1^ Triton X-100. The elution of the retained enzyme activities was accomplished at ≈ 0.3 ml/min with a 70-ml linear gradient of 0–400 mM NaCl in the washing buffer. Fractions of ≈ 2 ml were collected and phosphomonoesterase or phosphodiesterase activities on several substrates were assayed (see below). A 2-ml fraction (with V_e_ ≈ 40 ml) containing 48 μg protein ml^-1^ and the maximal activities on c-di-AMP and bis-4-nitrophenylphosphate was chosen for further fractionation by preparative electrophoresis.

One milliliter of the fraction with maximal activity was concentrated by putting it in a dialysis bag under solid sucrose for 14 h at 4°C, and the resulting sample was dialyzed for 5 h against 100 ml of 62 mM Tris-HCl, pH 6.8 at 25°C, 20 g l^-1^ sodium dodecyl sulfate, 100 g l^-1^ glycerol, and 25 mg l^-1^ bromophenol blue. The final sample volume was 134 μl, which was supplemented with 85 mM dithiothreitol. After 5 min at 37°C, the sample was submitted to electrophoresis on a 1.5-mm thick, 9% polyacrylamide gel containing sodium dodecyl sulfate [36], by application of 24 μg protein per lane. Electrophoresis was run at 30 mA for 60 min. One lane loaded with sample was cut out from the unstained gel and divided in 2.5 mm pieces along the electrophoretic axis. Each piece was washed in water and crushed in 0.3 ml 50 mM Tris-HCl, pH 7.5, with 2 mg ml^-1^ bovine serum albumin. After 14 h at 4°C, phosphodiesterase activities on c-di-AMP, c-di-GMP and bis-4-nitrophenylphosphate were assayed (see below). All the activities appeared in a single gel piece centered at about 15 mm of the electrophoretic origin. The rest of the polyacrylamide gel was stained for 20 min with 1 g l^-1^ Coomassie blue in 50% methanol and 10% acetic acid (by volume), followed by destaining in 10% methanol. By correlating enzyme activities with protein banding, a ≈ 66-kDa band was chosen for peptide-mass fingerprinting.

### Peptide mass fingerprinting and Mascot search

The stained protein band of ≈ 66 kDa that correlated with cyclic diadenylate phosphodiesterase activity after electrophoresis was cut out from the gel and processed for peptide mass fingerprinting by tryptic digestion in the Unidad de Proteómica of the Facultad de Farmacia, Universidad Complutense, Madrid (https://www.ucm.es/gyp/proteomica). The analysis by MALDI-TOF mass spectrometry yielded 65 mass signals observed between 850 Da and 3260 Da. Peptide mass fingerprint searches were run with these data in the Mascot Server of Matrix Science (http://www.matrixscience.com/server.html) assuming cysteine carbamidomethylation as fixed and methionine oxidation as variable modifications, and with a peptide mass tolerance of 50 ppm.

### Genomic DNA from *E*. *coli* BL21 cells

An aliquot of BL21 cells was seeded onto a LB agar plate (14 g l^-1^ bacto-agar, 10 g l^-1^ bacto-tryptone, 5 g l^-1^ yeast extract, 10 g l^-1^ NaCl). One colony was picked and inoculated in 10 ml of liquid LB medium. After 13 h at 37°C with shaking, 1.3 ml of the culture was centrifuged for 5 min at 10000 × g to collect the cell precipitate, from where genomic DNA was purified using the Wizard Genomic DNA kit (Promega) following the steps indicated by the supplier for Gram-negative bacteria. In summary: cell lysis, ribonuclease treatment, protein precipitation, isopropanol precipitation of DNA, washing with ethanol, and rehydrating. DNA concentration was estimated taking into account that one unit *A*_260_ corresponds to 50 μg ml^-1^.

### PCR amplification of the coding sequence of mature CpdB

The experimental design was based on the sequence with accession number NC_012892 (4347975..4349918, complement), which corresponds to the 1944-nt coding sequence of the *cpdB* gene of *E*. *coli* BL21 (DE3) and encodes a protein of 647 amino acids (accession number WP_000589409). The sequence of the 46-nt forward primer (CpdB-Fow) was CACTGGGGATCCGCGACaGTCGATCTACGTATCATGGAAACCACTG. It contains nucleotides 58–91 of the *cpdB* coding sequence (except for a silent substitution, G63A, introduced to optimize the primer; lowercase), immediately preceded by a 5´ extension of 12 nt that includes a BamHI site (underlined). The 49-nt reverse primer (CpdB-Rev) was CTGCACGAATTCTTACTTACTCAAATCCACCTGATAAATCGCAAACCCG. It contains the reverse complement of nucleotides 1908–1944 of the *cpdB* coding sequence, immediately preceded by a 5´ extension of 12 nt that includes an EcoRI site (underlined). The amplicon was expected to contain, between the designed BamHI and EcoRI sites, the coding sequence of mature CpdB (628 amino acids).

The PCR reaction mixture contained, in a volume of 50 μl, 160 ng of BL21 genomic DNA, 0.3 μM each of CpdB-Fow and CpdB-Rev (Invitrogen), 0.2 mM each of the four standard deoxynucleoside triphosphates (Roche), 1 μl of the Advantage cDNA polymerase mix and the recommended buffer (Clontech). A touchdown strategy was used, including an initial 2-min incubation at 95°C, 5 cycles of 30 s denaturation at 95°C, 1 min annealing at 62°C and 4 min extension at 72°C, another 5 cycles with annealing at 59°C and 25 cycles with annealing at 56°C, a final 7 min incubation at 72°C and rapid cooling to 4°C. Agarose gel electrophoresis analysis showed a single major band in very good agreement with the expected size of 1911 pb.

### Construction of plasmid pGEX-6P-3-cpdB

Since the products of the Advantage cDNA polymerase mix are expected to contain 3´ A extensions, and the electrophoretic analysis of the PCR reaction showed a strongly predominant product of the expected size, the reaction mixture was purified using the High Pure PCR Product Purification Kit (Roche) and was directly used for ligation with T4 DNA ligase to the linearized pGEM-T Easy vector that contains 3´ T extensions (pGEM-T Easy Vector System I; Promega). The ligation mixture (20 μl) was used to transform 40 μl of competent JM109 cells (Promega) by incubation for 30 min on ice, 45 s at 42°C and 2-min cooling on ice. The cells were diluted with 300 μl of LB medium, shaken for 60 min at 37°C, and seeded on LB-agar plates supplemented with 100 mg l^-1^ ampicillin, over which 100 μl of 100 mM isopropyl β-D-1-thiogalactopyranoside (IPTG; Roche) and 20 μl of 50 mg ml^-1^ 5-bromo-4-chloro-3-indolyl β-D-galactopyranoside (X-Gal; Roche) were spread before use. White colonies, which were expected to contain plasmid with insert disrupting the β-galactosidase coding sequence of the vector, were selected and their plasmids (High Pure Plasmid Isolation Kit, Roche) were analyzed by DNA sequencing from both ends of the T/A cloning site (Sp6 and T7 sequencing primers). One plasmid (pGEM-T-Easy-cpdB) which contained the full coding sequence of mature CpdB confirmed by double-strand sequencing (Servicio de Genómica, Instituto de Investigaciones Biomédicas “Alberto Sols”, Consejo Superior de Investigaciones Científicas, Universidad Autónoma, Madrid) was selected for further work. These sequence data have been submitted to the GenBank database under accession number KP938772.

The expression plasmid pGEX-6P-3-cpdB was constructed by directionally subcloning the BamHI-EcoRI insert obtained from pGEM-T-Easy-cpdB between the corresponding sites of plasmid pGEX-6P-3 (GE Healthcare Life Sciences), and transforming JM109 cells with the ligation mixture. Colonies were obtained over LB-agar plates supplemented with 100 mg l^-1^ ampicillin and selected by sequencing from both ends of the insertion site with pGEX-5 and pGEX-3 sequencing primers.

### Expression and purification of recombinant CpdB from pGEX-6P-3-cpdB

Plasmid pGEX-6P-3-cpdB contains the mature CpdB coding sequence in frame with the coding sequence of the glutathione S-transferase (GST) label included in the vector, and separated from it by the PreScission protease cut sequence and the BamHI site. Therefore, mature CpdB can be expressed from the IPTG-inducible tac promoter of pGEX-6P-3-cpdB as a GST fusion protein which can be cut with PreScission (GE Healthcare Life Sciences), yielding mature CpdB with a N-terminal extension of 5 amino acids (GPLGS).

One BL21 colony transformed with pGEX-6P-3-cpdB and selected by ampicillin resistance was inoculated in 10 ml of LB medium supplemented with 100 mg l^-1^ ampicillin. This culture, after overnight shaking at 37°C, was diluted with 200 ml of the same medium and it was further incubated at 30°C until *A*_600_ reached a value of ≈ 0.8. At this point, IPTG was added to a final concentration of 0.5 mM. After an additional 2–3 h incubation, when *A*_600_ reached a value of ≈ 1.4, cells were collected by centrifugation for 15 min at 4000 × g and 4°C. The precipitate was resuspended in 20 ml of 20 mM Tris-HCl, pH 7.5 at 4°C, 0.5 mM EDTA, 1 mM dithiothreitol, 50 mM NaCl, supplemented with two tablets of protease inhibitor cocktail (Complete Mini EDTA Free, Roche). Then, the cells were lysed by sonication (see above). The lysate was fractionated by centrifugation for 30 min at 85000 × g and 4°C. The supernatant and the precipitate, the latter resuspended in 20 ml of lysis buffer, were frozen at –20°C until used.

For purification of recombinant CpdB, 20 ml of thawed supernatant were applied at 0.1 ml/min to a 2-ml glutathione (GSH)-Sepharose column (GE Healthcare Life Sciences) equilibrated with lysis buffer. Unretained proteins were washed out with 40 ml of 50 mM Tris-HCl, pH 7.5 at 4°C, 1 mM EDTA, 1 mM dithiothreitol, 150 mM NaCl. After this, 80 units of PreScission protease was applied to the column in 2 ml of wash buffer, and flow was detained for 16 h to allow for in-column proteolysis of the recombinant GST-CpdB fusion protein at 4°C. Recombinant CpdB with the N-terminal GPLGS extension, but separated from the GST tag, was collected in a few fractions when the chromatographic flow was resumed with the same elution buffer. In the purified preparation, protein content [35] and enzyme activities (see below) were assayed.

The protein analysis of the expression and purification steps was performed by denaturing gel electrophoresis as described above for soluble lysates of *E*. *coli*, except that (i) protein samples of 14 μl mixed with 2 μl of 1 M dithiothreitol and with 4 μl of 310 mM Tris-HCl, pH 6.8 at 25°C, 100 g l^-1^ sodium docecyl sulfate, 500 g l^-1^ glycerol, and 125 mg l^-1^ bromophenol blue, were incubated for 2 min at 100°C before application to the gel, and (ii) the gel was stained for 60 min and destained in 10% methanol and 10% acetic acid (by volume).

### Enzyme activity assays

All the activities assayed correspond to phosphohydrolytic reactions in which either phosphomonoester, phosphodiester or phosphoanhydride linkages are hydrolyzed. The hydrolysis of 2´-AMP, 3´-AMP, 5´-AMP, ATP, 4-nitrophenylphosphate (all from Sigma) and ADP (Roche), was estimated from the amount of phosphate directly formed as product by the enzyme being assayed (i.e. CpdB), as measured by a sensitive colorimetric assay (see below). The hydrolysis of 2´,3´-cAMP, 2´,3´-cCMP, 2´,3´-cUMP, 2´,3´-cGMP, 3´,5´-cAMP, bis-4-nitrophenylphosphate, Ap_4_A, Ap_3_A, ADP-ribose, CDP-choline, ADP-glucose and UDP-glucose (all from Sigma), were similarly assayed but with the inclusion of an excess of alkaline phosphatase in the reaction mixture, which liberated phosphate from the reaction products of the enzyme being assayed. The hydrolysis of pApA, pGpG, c-di-AMP and c-di-GMP (all from Biolog) were assayed by HPLC measuring the formation of 5´-AMP or 5´-GMP as products (see below).

Unless otherwise indicated, the reaction mixtures contained 50 mM Tris-HCl, pH 7.5 at 37°C, 2 mM MnCl_2_, 6.5 units ml^-1^ of alkaline phosphatase (only when needed, see above; Roche), 0.1 mg ml^-1^ bovine serum albumin (Roche), and variable amounts of the enzyme sample to be assayed. The reactions were initiated by addition of substrate (final concentration 0.75 mM or as indicated) after a 5-min preincubation of the reaction mixture at 37°C. Enzyme incubations were terminated either by addition of a phosphate reagent (see below) or by injection in a HPLC column (see below). All the assays were run at 37°C, under conditions of linearity with respect to incubation time and enzyme amount. Controls without enzyme and/or substrate were run in parallel to full reaction mixtures. One unit enzyme activity (U) is defined as 1 μmol min^-1^.

### Inorganic phosphate assay

This assay was applied to measure enzyme activities (see above). Depending on the sensitivity needed, two different versions of the Ames reagent [37] were used. The normal reagent was prepared mixing 6 volumes of 3.4 mM ammonium heptamolybdate in 0.5 M H_2_SO_4_, 1 volume of 570 mM ascorbic acid and 1 volume of 130 mM sodium dodecyl sulfate. In most cases, 350–700 μl of this reagent was added over 50–100 μl samples taken from enzyme reaction mixtures or their controls. Nevertheless, if higher sensitivity was needed, a concentrated reagent was used that contained 5 volumes of 13.8 mM ammonium heptamolybdate in 2 M H_2_SO_4_, 3 volumes of 570 mM ascorbic acid and 1.1 volume of 350 mM sodium dodecyl sulfate. In this case, 200 μl of the concentrated reagent was added over 400 μl samples. In all the assays, *A*_820_ was measured after 20 min at 45°C and cooling to ambient temperature. Inorganic phosphate content in the samples was calculated using a standard curve with known phosphate amounts.

### HPLC analysis of reaction products

This technique was routinely used to assay the activities on linear and cyclic dinucleotides, and also to establish the product pattern of hydrolytic reactions of 2´,3´-cAMP, ATP and ADP. The separations were accomplished with a Tracer Excel 120 column (150 mm × 4 mm) protected by a pre-column (10 mm × 4 mm) of the same material (octadecylsilica; Teknokroma, San Cugat del Vallés, Barcelona). An ion-pair reverse phase strategy was used in a HP1100 system with a diode array detector adjusted to measure *A*_260_. Samples of 20 μl were injected and the elution was performed at 1 ml/min with two buffers: A, 5 mM sodium phosphate, pH 7.0, 5 mM tetrabutylammonium, 20% methanol (by volume); B, 100 mM sodium phosphate, pH 7.0, 5 mM tetrabutylammonium, 20% methanol. The initial mobile phase and the gradient method depended on the enzymatic reaction being studied, as indicated below.

Method 1, used to analyze the hydrolysis of 2´,3´-cAMP. Initial mobile phase: 100% A. Gradient: linear up to 100% B in 10 min.

Method 2, used to analyze the hydrolysis of pApA, ATP and ADP. Initial mobile phase: 80% A, 20% B. Gradient: linear up to 100% B in 10 min.

Method 3, used to analyze the hydrolysis of c-di-AMP. Initial mobile phase: 100% A. Gradient: linear up to 60% B in 5 min, and then linear up to 70% in another 10 min.

Method 4, used to analyze the hydrolysis of c-di-GMP. Initial mobile phase: 80% A, 20% B. Gradient: linear up to 60% B in 10 min.

Method 5, used to analyze the hydrolysis of pGpG. Initial mobile phase: 100% A. Gradient: linear up to 60% B in 10 min, and then linear up to 70% in another 5 min.

### Estimation of enzyme kinetic parameters

The parameters *k*_cat_ and *K*_m_ were estimated from saturation kinetics experiments in which initial rates of hydrolysis were measured at different substrate concentrations. The Michaelis-Menten equation was adjusted to the rate-versus-substrate concentration datapoints of each experiment by nonlinear regression using the Solver tool of Microsoft Excel 2011 for the Mac version 14.1.0. A least squares fitting was applied and V_max_ (convertible to *k*_cat_) and *K*_m_ were allowed to fluctuate in the adjustment.

## Results

### Identification of CpdB as the *E*. *coli* protein active as cyclic diadenylate phosphodiesterase

**Fig 1:**
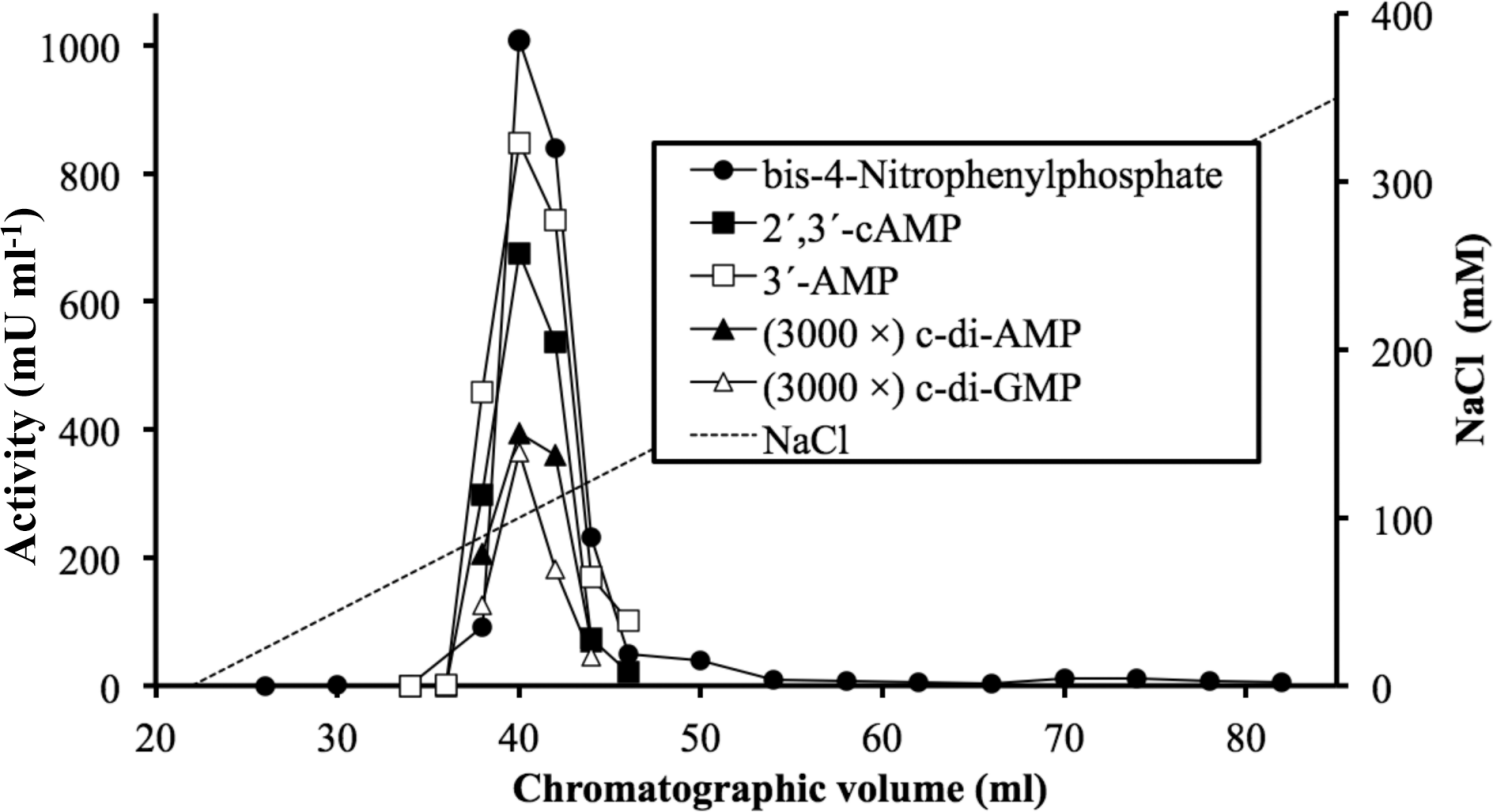
Ion-exchange chromatography of the cyclic diadenylate phosphodiesterase present in soluble lysates of *E*. *coli* BL21: coincidence with phosphohydrolytic activities over other substrates. This corresponds to the second step of the purification, where a sample obtained by gel-filtration chromatography was fractionated by chromatography on a Q-Sepharose column. This experiment was performed when the protein associated with the activities have been already identified as CpdB by peptide mass fingerprinting. In the graph, the activities on cyclic dinucleotides are multiplied by 3000.

**Fig 2:**
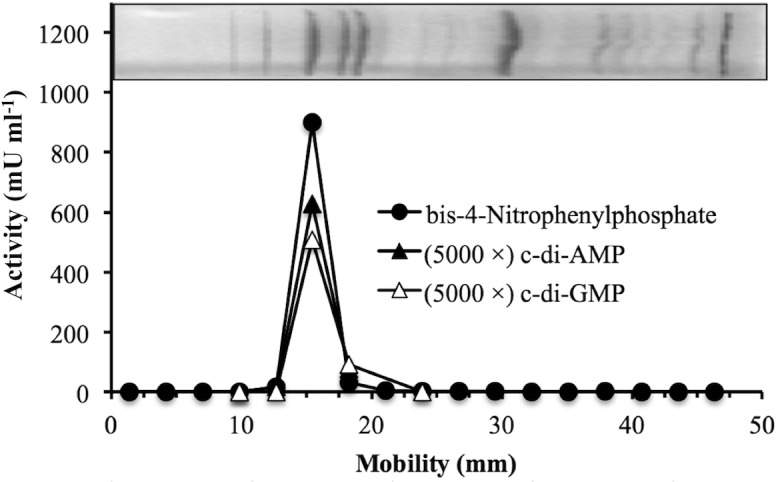
Identification of the endogenous cyclic diadenylate phosphodiesterase. Correlation of cyclic diadenylate phosphodiesterase activity with a ≈ 66-kDa protein band. Two identical lanes of the same denaturing electrophoresis gel were loaded each with 24 μg of partially purified cyclic diadenylate phosphodiesterase (Fig 1). One of them was stained with Coomassie blue (top), and the other was cut into pieces and extracted under conditions that favored renaturation. In the extracts of these pieces, the phosphohydrolytic activities on the indicated substrates were assayed. They are plotted in the main graph. The activities on cyclic dinucleotides are multiplied by 5000. The protein band coinciding with the activities was processed to obtain its tryptic fingerprint. Out of 65 peptide masses recorded, 21 coincided with tryptic peptides of CDPB_ECOLI with a 50 ppm tolerance.

A soluble lysate of nontransformed *E*. *coli* BL21 cells was fractionated by gel filtration and ion-exchange chromatography, what yielded a partially purified preparation enriched in c-di-AMP phosphodiesterase activity (Fig 1). Subsequent experiments showed that the same chromatographic fractions contained coeluting activities on c-di-GMP, 3´-AMP, 2´,3´-cAMP and bis-4-nitrophenylphosphate (Fig 1). Coomassie blue-stained SDS-PAGE gels showed that the partially purified preparation contained multiple protein bands. In a parallel, non-stained lane, the gel was cut into pieces that were crushed and extracted. Phosphodiesterase activity on cyclic dinucleotides and on bis-4-nitrophenylphosphate correlated with a ≈ 66-kDa protein band (Fig 2). A Mascot search was performed in the Matrix Science web site (http://www.matrixscience.com), querying the SwissProt database with the data of a tryptic peptide-mass fingerprint of the ≈ 66-kDa protein. A single candidate was found above the threshold of significance (Mascot score 70). This was the protein named CPDB_ECOLI (Mascot score 169), from the K12 strain of *E*. *coli*, with a molecular mass of 70.90 kDa, labeled as a 2´,3´-cyclic nucleotide phosphodiesterase and 3´-nucleotidase. It is encoded by the *cpdB* gene as the precursor of a periplasmic protein composed by 647 amino acids, including a 19 amino acid signal sequence in the N end [1]. It corresponds to the 3´-nucleotidase / 2´,3´-cyclic-nucleotide phosphodiesterase early studied in bacterial extracts [2, 3], but so far not expressed and characterized enzymatically as a recombinant protein. Its possible activity as cyclic dinucleotide phosphodiesterase was unknown.

The sequenced genome of *E*. *coli* BL21 (DE3) contains a *cpdB* gene (GenBank accession number NC_012892: 4347975..4349918, complement) which codes also for a 647 amino acid protein (accession number WP_000589409) with 641 identical amino acids to the CPDB_ECOLI protein (accession number P08331) of *E*. *coli* K12. Bioinformatic analysis of the full translation of BL21 *cpdB* confirmed the presence of a N-terminal signal peptide of 19 amino acids, expected to be excised during secretion to the periplasmic space [38]. This would produce a 628 amino-acid mature protein of 68.85 kDa, very near to the ≈ 66 kDa of the protein band associated with the endogenous c-di-AMP phosphodiesterase of BL21 cells (Fig 2). Therefore, the coding sequence of mature CpdB was amplified by PCR from BL21 genomic DNA, as described under Materials and Methods. The resulting sequence has been submitted to the GenBank database under accession number KP938772. Its theoretical translation, with accession number AKS04560, was identical to the one deduced from the *E*. *coli* BL21 (DE3) genome except for a Gln>Arg substitution at position 409 (i.e. position 428 of precursor protein).

**Fig 3:**
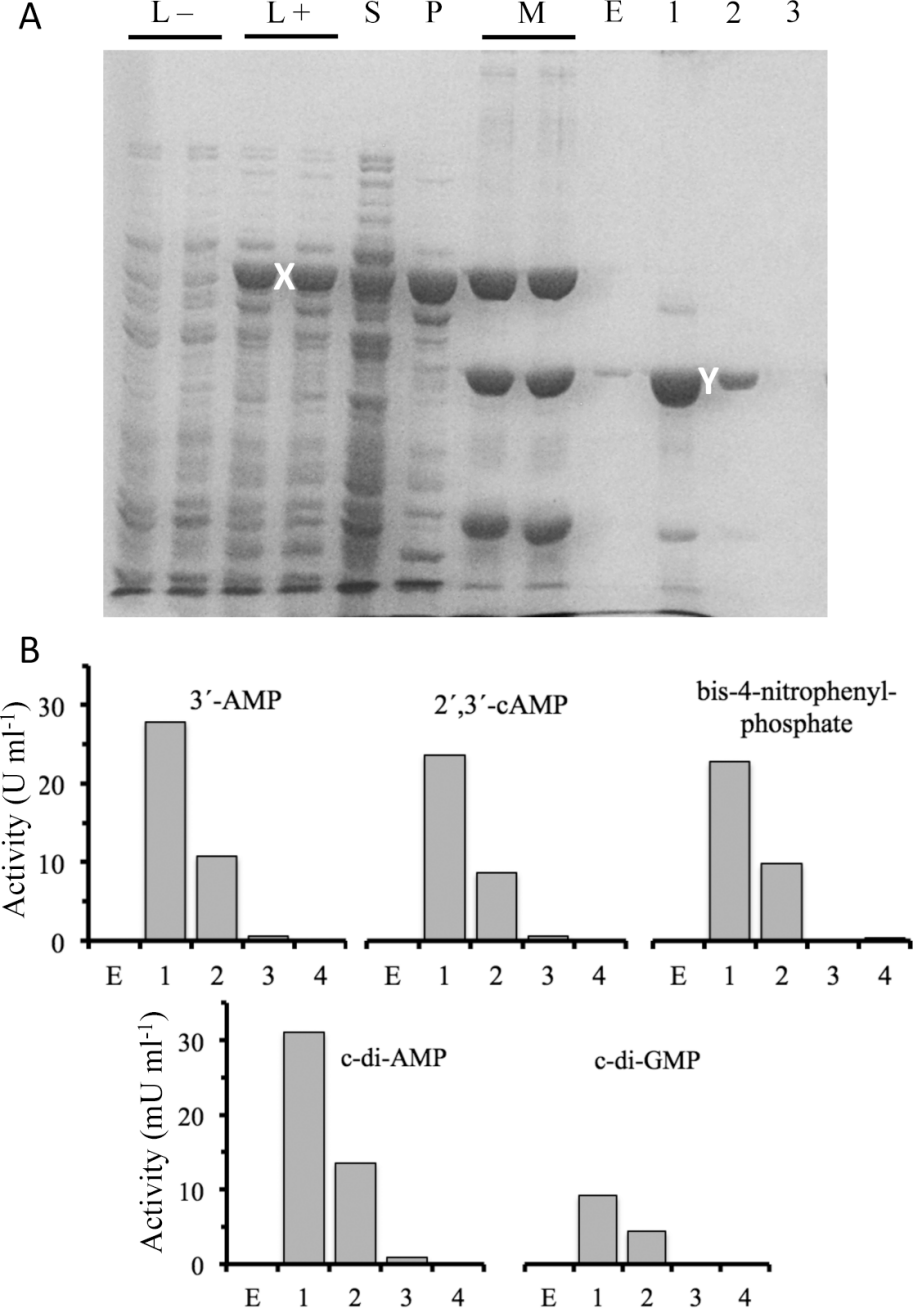
Expression and purification of mature CpdB. The recombinant protein was expressed in BL21 cells transformed with pGEX-6P-3-cpdB and induced by IPTG. The GST-CpdB fusion protein was purified by affinity adsorption to GSH-Sepharose. Mature CpdB separated from the GST tag was recovered from the gel after in-column digestion with PreScission. (A) Denaturing gel electrophoresis analysis. L–and L+, bacterial lysates before and after IPTG induction. S and P, supernatant and precipitate of the L+ lysate. M, molecular weight markers (phosphorylase B, 97400; bovine serum albumin, 66200; ovoalbumin, 45000). E, fraction excluded from the GSH-Sepharose column during application of the PreScission protease. 1–4, fractions collected after intracolumn proteolysis. X and Y, bands corresponding to the GST-CpdB fusion and to GPLGS-CpdB obtained by proteolysis. (B) Phosphohydrolytic activities on the indicated substrates, measured in the fractions collected from the GSH-Sepharose column.

The mature form of CpdB was overexpressed as a fusion protein with glutathione S-transferase (GST) in *E*. *coli* BL21 cells transformed with plasmid pGEX-6P-3-cpdB and induced with IPTG. The recombinant protein showed the expected size of ≈ 96 kDa. After affinity adsorption to GSH-Sepharose followed by in-column specific proteolysis with PreScission, it was recovered as a ≈ 69-kDa protein (88% pure), in agreement with the size expected for mature CpdB with a GPLGS N-terminal extension. The two fractions where the protein was recovered contained also activities that correlated with the intensity of the ≈ 69-kDa protein band, including c-di-AMP phosphodiesterase (Fig 3). The pooled fractions constituted the preparation used for the enzymatic characterization of CpdB. The enzyme was active as a monomer, since its native molecular mass determined by gel-filtration chromatography was ≈ 65 kDa.

### Enzymatic characterization of recombinant CpdB

**Fig 4:**
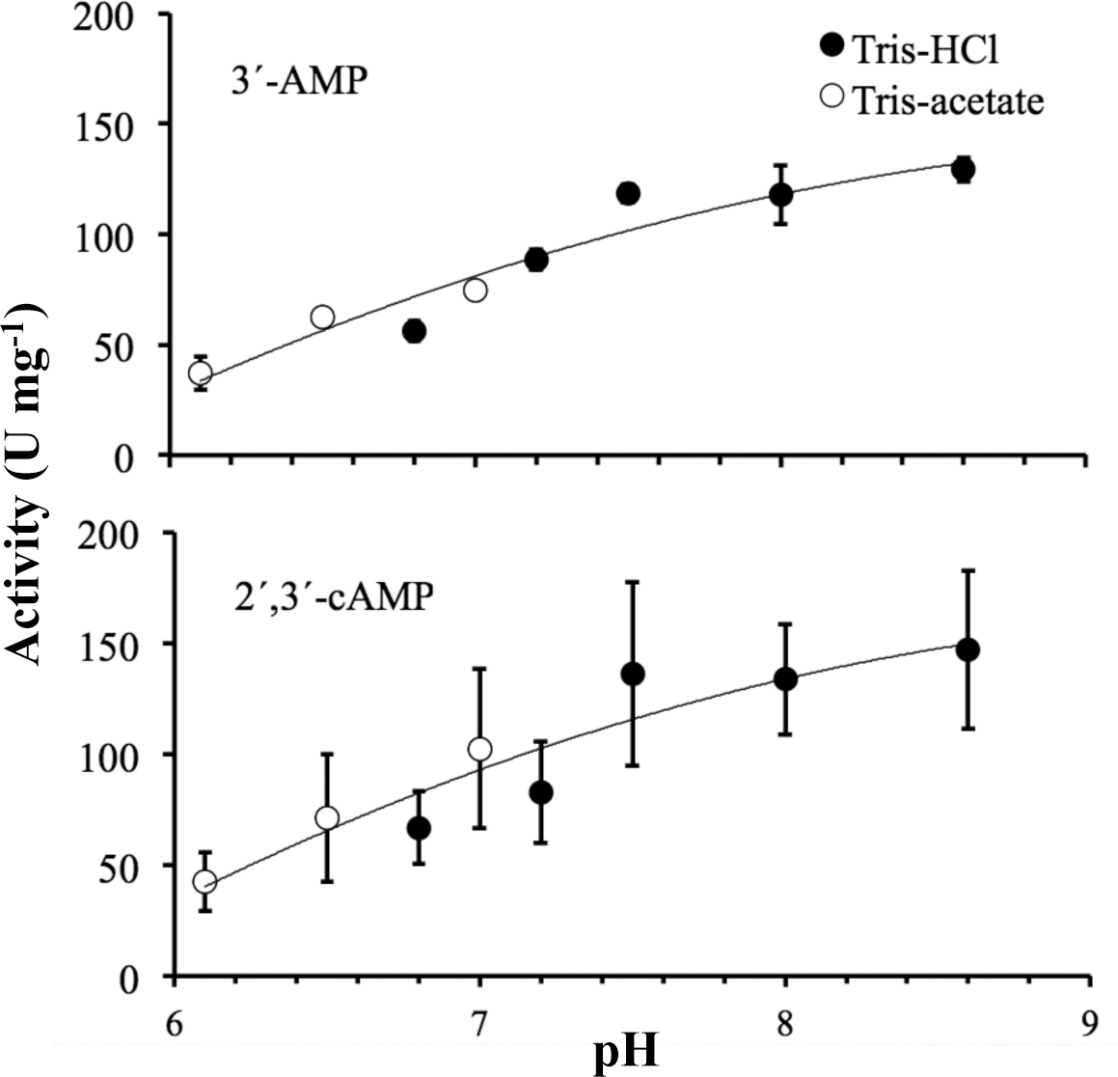
Response of recombinant CpdB activities to pH. The activities were assayed on the indicated substrates under standard conditions except that 100 mM Tris-HCl or 100 mM Tris-acetate were used as buffers. The values of pH were taken directly from reaction mixtures at 37°C. Each data point is a mean value ± standard deviation (n = 3).

**Fig 5:**
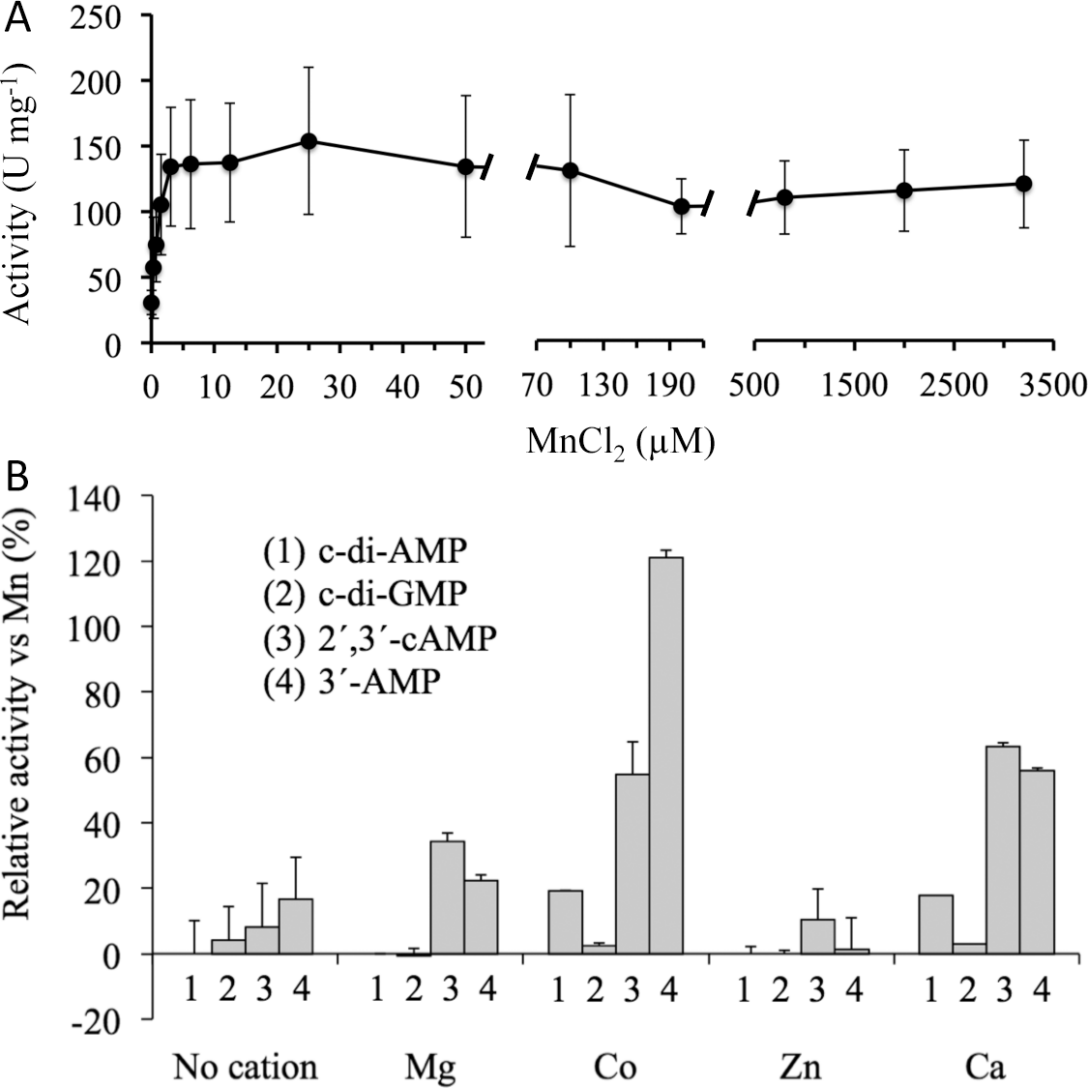
Response of recombinant CpdB activities to divalent cations. (A) Response of the phosphohydrolytic activity on 2´,3´-cAMP to varying concentration of Mn^2+^. Data points are mean values ± standard deviations of 3–6 measurements in six different experiments. (B) Effects of cations alternative to Mn^2+^ as activators. In every case the cation was used at a 2 mM concentration of the chloride salt. Cyclic dinucleotides were used at 20 μM, 2´,3´-cAMP and 3´-AMP at 750 μM. The results are expressed as percentages of the activities measured with 2 mM Mn^2+^: c-di-AMP, 0.15 ± 0.015 U mg^-1^ (100 ± 10%); c-di-GMP, 0.05 ± 0.005 U mg^-1^ (100 ± 10%); 2´,3´-cAMP, 130 ± 17 U mg^-1^ (100 ± 13%); 3´-AMP, 120 ± 16 U mg^-1^ (100 ± 13%). The bars are mean values ± standard deviations of 3–5 measurements in five different experiments. All the differences with respect to the Mn^2+^-dependent controls were significant according to the Dunnett test (P < 0.01 or, in the case of the Co^2+^-dependent activity on 3´-AMP, P < 0.05).

Throughout this study, the enzymatic activities of CpdB were assayed at pH 7.5 in the presence of 2 mM MnCl_2_. These conditions were originally chosen by analogy to the c-di-AMP phosphodiesterase GdpP of *B*. *subtilis* [34] on which the first steps of this work were based (see the Introduction). Experiments performed after the identification of CpdB to test the effect of varying these conditions indicated that both the pH (Fig 4), and the nature and concentration of the divalent cation (Fig 5) were reasonable choices, so they were maintained.

The specificity of recombinant CpdB is summarized in Table 1 in terms of *k*_cat_, *K*_m_ and the catalytic efficiency parameter *k*_cat_/*K*_m_. Twenty compounds were hydrolyzed at detectable rates, although the activities covered a wide range. The measurable *k*_cat_ values of the reactions spanned 17000 fold from a highest 340 s^-1^ to a lowest 0.02 s^-1^, and *K*_m_ values spanned ≈ 8000 fold from 0.6 μM to 4600 μM. Accordingly, the catalytic efficiencies *k*_cat_/*K*_m_ spanned ≥200000 fold from a highest of 1.3 × 10^7^ M^-1^s^-1^ for 3´-AMP to less than 10^2^ M^-1^s^-1^ for ADP-glucose and UDP-glucose.

**Table 1:** Kinetic parameters of CpdB. The substrates are listed in order of decreasing catalytic efficiency (*k*_cat_/*K*_m_). The *k*_cat_ and *K*_m_ data are mean values ± standard deviations of 3–6 experiments.

Substrate^a^	*k*_cat_	*K*_m_	*k*_cat_/*K*_m_
	s^-1^	μM	M^-1^ s^-1^
3´-AMP	176 ± 20	14 ± 4	1.3 × 10^7^
2´,3´-cCMP	100 ± 20	12 ± 5	8.5 × 10^6^
2´,3´-cAMP	190 ± 20	27 ± 6	7.3 × 10^6^
2´,3´-cUMP	260 ± 20	62 ± 14	4.2 × 10^6^
bis-4-Nitrophenylphosphate	340 ± 80	96 ± 18	3.6 × 10^6^
2´,3´-cGMP	84 ± 9	25 ± 7	3.5 × 10^6^
pApA	1.3 ± 0.1	0.6 ± 0.1	2.3 × 10^6^
pGpG	0.08 ± 0.02	0.6 ± 0.6	4.0 × 10^5^
4-Nitrophenylphosphate	37 ± 5	183 ± 34	2.1 × 10^5^
ATP	12 ± 3	242 ± 39	4.8 × 10^4^
c-di-AMP	0.40 ± 0.17	15 ± 8	2.9 × 10^4^
Ap_4_A	0.86 ± 0.14	53 ± 9	1.6 × 10^4^
c-di-GMP	0.07 ± 0.01	6 ± 2	1.3 × 10^4^
Ap_3_A	0.66 ± 0.13	74 ± 24	9.2 × 10^3^
ADP	1.5 ± 0.3	203 ± 60	7.4 × 10^3^
ADP-ribose	0.19 ± 0.02	70 ± 12	2.8 × 10^3^
CDP-choline	0.51 ± 0.24	219 ± 99	2.3 × 10^3^
3´,5´-cAMP	0.56 ± 0.66	4600 ± 6400	2.2 × 10^2^
ADP-glucose	0.02 ± 0.01	304 ± 289	8.4 × 10^1^
UDP-glucose	0.02 ± 0.01	392 ± 121	2.8 × 10^1^

^a^ CpdB did not show detectable activity over 5´-AMP and 2´-AMP (see the text).

In the light of Table 1 data, CpdB is clearly a broad-specificity phosphohydrolase, but considering only its action on nucleoside monophosphates it behaved as a very specific 3´-nucleotidase. While 3´-AMP was the best substrate, with a very high catalytic efficiency, a distinctive feature of recombinant CpdB specificity was the absence of detectable activity over 5´-AMP and 2´-AMP. To judge from initial rate assays run at a fixed 750 μM substrate concentration, 5´-AMP and 2´-AMP were hydrolyzed, if something, at less than 25% of the hydrolytic rate of UDP-glucose (the worse measurably-hydrolyzed substrate) or less than 0.004% of 3´-AMP (the best substrate).

Just below the best substrate, in a second step of rather high catalytic efficiencies (10^6^−10^7^ M^-1^s^-1^), were the activities over 2´,3´-cyclic mononucleotides, the artificial phosphodiester bis-4-nitrophenylphosphate, and the linear dinucleotide pApA. In a third step, with moderate catalytic efficiencies (10^4^−10^6^ M^-1^s^-1^), were the linear dinucleotide pGpG, the phosphoric monoester 4-nitrophenylphosphate, the cyclic-dinucleotides c-di-AMP and c-di-GMP, the bis-5´,5´´´-dinucleoside oligophosphates Ap_4_A and Ap_3_A, and the adenosine 5´-oligophosphates ATP and ADP. Finally, with low catalytic efficiencies (10^2^−10^3^ M^-1^s^-1^) were ADP-ribose, CDP-choline, 3´,5´-cAMP, and with very low efficiencies (≤10^2^ M^-1^s^-1^) ADP-glucose and UDP-glucose.

All the reactions catalyzed by CpdB are of phosphohydrolytic character, either of phosphomonoesterase or phosphodiesterase type. Out of the substrates presented in Table 1, only the phosphomonoesters 3´-AMP and 4-nitrophenylphosphate display a single phosphoester linkage, allowing thus to infer the reaction pattern from the fact that CpdB catalyzes phosphate liberation directly: hence, 3´-AMP was hydrolyzed to adenosine and phosphate, and 4-nitrophenylphosphate to 4-nitrophenol and phosphate. The rest of the substrates contain two or more phosphoester or phosphoanhydride linkages and the expected pattern of reaction depends on the identity of the linkage hydrolyzed. Most of the compounds tested as substrates are devoid of terminal phosphoryl groups, and they are not susceptible to alkaline phosphatase activity. This allowed the use of a phosphomonoesterase-coupled assay to monitor the hydrolysis by CpdB of phosphatase-resistant substrates to phosphatase-susceptible products, with the concomitant liberation of phosphate. However, this assay did not distinguish among the possible phosphohydrolytic patterns. The ambiguity was resolved for a selection of the substrates as follows.

**Fig 6:**
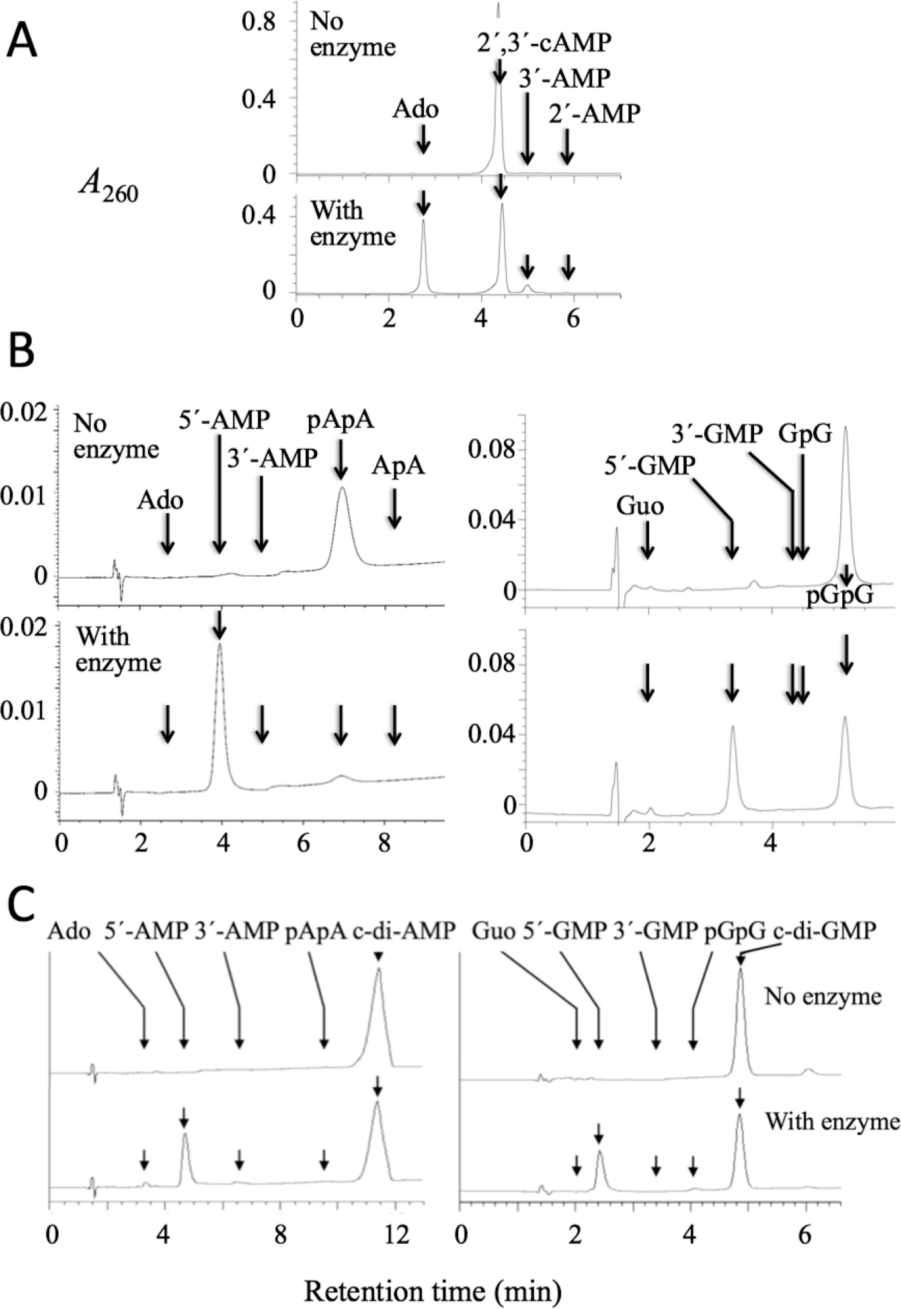
Reaction products of recombinant CpdB by HPLC. All the incubations were performed at 37°C. (A) Hydrolysis of 500 μM 2´,3´-cAMP by 0.23 μg ml^-1^ of CpdB in a 12-min incubation. (B) Hydrolysis of (left panels) 10 μM pApA by 1.5 μg ml^-1^ of CpdB in a 4-min incubation, or (right panels) 5 μM pGpG by 1 μg ml^-1^ of CpdB in a 20-min incubation. (C) Hydrolysis of (left panels) 20 μM c-di-AMP by 0.3 μg ml^-1^ of CpdB in a 15-min incubation, or (right panels) 20 μM c-di-GMP by 15 μg ml^-1^ of CpdB in an 8-min incubation.

The hydrolysis of the cyclic phosphodiester 2´,3´-cAMP by CpdB, in the absence of alkaline phosphatase, was monitored by HPLC under conditions which resolved the substrate from the two possible primary products 3´-AMP and 2´-AMP, and from the secondary product adenosine. The results showed that the consumption of 2´,3´-cAMP was accompanied by the formation of adenosine and a minor amount of 3´-AMP. The formation of 2´-AMP was not detectable. Since the latter is not a CpdB substrate, whereas 3´-AMP is a very good one (Table 1), it was concluded that the enzyme hydrolyzed the 2´-phosphoester linkage of 2´,3´-cAMP, and the 3´-AMP formed was rapidly hydrolyzed to adenosine (Fig 6A). The strong specificity of CpdB for the 2´-phosphoester linkage of 2´,3´-cAMP is in marked contrast with the strong specificity for the hydrolysis of 3´-AMP not 2´-AMP as far as the non-cyclic nucleotides are concerned.

The hydrolysis of the linear dinucleotides pApA and pGpG, which contain a 5´ phosphomonoester and a 3´-5´ phosphodiester linkage, were also monitored by HPLC under conditions which resolved the substrate from possible primary or secondary products ApA (GpG), 3´-AMP (3´-GMP), 5´-AMP (5´-GMP) and adenosine (guanosine). The results showed the direct conversion of substrates to the corresponding 5´-nucleotide, without traces of the other possible products. It was concluded that CpdB hydrolyzed the phosphodiester linkage on its 3´ side (Fig 6B).

The hydrolysis of the cyclic dinucleotides c-di-AMP and c-di-GMP, which contain two 3´-5´ phosphodiester linkages, were also monitored by HPLC under conditions which resolved the substrate from possible primary or secondary products. The results showed the direct conversion of substrates to the corresponding 5´-nucleotide, without significant traces of the other possible products. It was concluded that CpdB hydrolyzed one of the 3´-5´ phosphodiester linkages on its 3´ side, and that the linear dinucleotide formed was rapidly hydrolyzed to the corresponding 5´-nucleotide (Fig 6C).

The hydrolysis of ATP and ADP was routinely assayed by measuring phosphate liberation but the reaction pattern was also assayed by HPLC. The hydrolysis of ADP gave 5´-AMP as the only detectable product. The hydrolysis of ATP yielded ADP as product, and 5´-AMP formation was detected only after considerable accumulation of ADP.

## Discussion

### Comparison of the efficiency and specificity of the 3´-nucleotidase CpdB to the 5´-nucleotidase UshA, both present in the periplasm of *E*. *coli*, and their role in the assimilation of exogenous nucleotides

To our knowledge, the catalytic efficiency of CpdB was here quantitated for the first time. The *k*_cat_/*K*_m_ value >10^7^ M^-1^s^-1^ for the hydrolysis of 3´-AMP (Table 1), represents a very high catalytic efficiency, not far from the value of 10^8^−10^9^ M^-1^s^-1^ that corresponds to the theoretical maximum of catalytic activity determined by the diffusion-controlled encounter of the enzyme and substrate [39, 40]. In this respect, and in its structure (see below), CpdB resembles UshA, also a highly-efficient periplasmic phosphohydrolase of *E*. *coli* that hydrolyzes 5´-AMP with *k*_cat_/*K*_m_ ≈10^8^ M^-1^s^-1^ [41]. Such very high catalytic efficiencies are not exceptional in the enzyme universe, but neither are they very common. In a survey of the catalytic efficiencies recorded in databases for almost two-thousand enzymes, only 7% of them showed catalytic efficiencies ≥10^7^ M^-1^s^-1^ [42].

CpdB is a wide-specificity phosphohydrolase but, as far as simple, canonical nucleotides are concerned, the preference for 3´-nucleotides over their 5´- or 2´- counterparts is noteworthy. This was early described for enzyme purified from bacterial extracts by Anraku, who reported activities on 5´-AMP and 2´-AMP of 3–10% of that on 3´-AMP [2, 3]. In our conditions, the preference of recombinant CpdB for the 3´-phosphomonoester linkage was even stronger than that (see under Results). On the other hand, UshA is also a wide-specificity hydrolase but, concerning simple nucleotides, it displays strict specificity for 5´-AMP as it does not hydrolyze 3´-AMP nor 2´-AMP [41, 43, 44]. Other major differences between UshA and CpdB concern their activities (i) on 2´,3´-cyclic nucleotides, which are not hydrolyzed by UshA [41, 43] but are excellent substrates for CpdB [2, 3], just below 3´-AMP in catalytic efficiency (Table 1), and (ii) on UDP-sugars or CDP-alcohols, which are excellent UshA substrates [43, 45] with efficiencies similar to 5´-AMP [41] but are only weak substrates of CpdB (Table 1).

The *cpdB* and *ushA* genes are both involved in the bacterial assimilation of exogenous nucleotidic compounds which cannot be transported through the cell membrane, whereas their degradation products nucleoside and phosphate can. Evidence for this role has been obtained by study of *cpdB* or *ushA* mutants. For instance, *cpdB* mutants of an *E*. *coli* strain deficient in the synthesis of pyrimidines (*pyrE* mutants) are unable to use 2´,3´-cUMP as sole pyrimidine source, in contrast to *cpdB*^+^ *pyrE* cells [46]; the *cpdB* mutants of *Y*. *enterocolitica* do not grow on 2´,3´-cAMP as sole source of carbon and energy [4]; and the *cpdB* mutants of *V*. *cholerae* showed a greatly reduced growth with 3´-AMP as the sole source of phosphate [5]. Similar experiments performed with *ushA* mutants, indicate that UshA is required for growth of *E*. *coli* with 5´-AMP as the sole carbon source [47–49]. The location of mature CpdB and UshA proteins in the periplasmic space of Gram-negative cells, their complementary specificities, and their very high catalytic efficiencies on the respective 3´- or 5´-nucleotide substrates, make them a well-suited enzymatic equipment for nucleotide assimilation. Indeed, the joint role of both enzymes in the growth of bacteria supported by extracellular DNA as source of carbon and phosphorus has been very recently demonstrated in *V*. *cholerae* [5]. The notion that CpdB and UshA may work as a team for this task is highlighted by the occurrence of YfkN, a protein encoded by a natural fusion of *cpdB* and *ushA* genes in *B*. *subtilis* [50].

### Several comparatively-minor substrates of CpdB, including cyclic and linear dinucleotides, are hydrolyzed with catalytic efficiencies typical of the ‘average’ enzyme

Compared to 3´-nucleotides and 2´,3´-cyclic nucleotides, hydrolyzed with *k*_cat_/*K*_m_ values of 10^6^−10^7^ M^-1^s^-1^, most of the other CpdB substrates may be deemed to represent minor ones in relative terms (Table 1). However, it must be remarked that several of these CpdB substrates were hydrolyzed with substantial efficiencies in absolute terms. This is the case of the linear dinucleotides pApA, pGpG, the cyclic-dinucleotides c-di-AMP and c-di-GMP, the bis-5´,5´´´-dinucleoside oligophosphates Ap_4_A and Ap_3_A, and the adenosine 5´-oligophosphates ATP and ADP, hydrolyzed by CpdB with *k*_cat_/*K*_m_ values of 10^4^−10^6^ M^-1^s^-1^ (Table 1). For comparison, it can be recalled that the median of the distribution of catalytic efficiencies in the enzyme universe lies at ≈10^5^ M^-1^s^-1^, and 60% of the enzymes with known catalytic efficiencies fall in the 10^4^−10^6^ M^-1^s^-1^ range [42]. Therefore, the mentioned activities of CpdB, far from negligible, are quantitatively like that of the ‘average’ enzyme. This level of activity cannot be disregarded, particularly because CpdB is active in the periplasmic space of Gram-negative bacteria, where the access to different kinds of nucleotides depends on their presence in the environment, not necessarily in the intracellular medium of the bacteria. It is thus quite reasonable to assume that, at least in the absence of the major CpdB substrates, 3´-nucleotides and 2´,3´-cyclic nucleotides, substrates that are hydrolyzed with efficiencies typical of the ‘average’ enzymes would be amenable to hydrolysis. In this concern, we think that the cyclic dinucleotide phosphodiesterase activity of CpdB is worthy of particular attention because (i) its catalytic efficiency is comparable to that of known cyclic dinucleotide phosphodiesterases, (ii) the *cpdB* gene has effects on bacterial virulence, a phenotype which is frequently modulated by cyclic dinucleotides, and (iii) the other cyclic dinucleotide phosphodiesterases are cytoplasmic while CpdB is periplasmic (see below).

### Comparison of CpdB to known, bona fide cyclic dinucleotide phosphodiesterases

**Fig 7:**
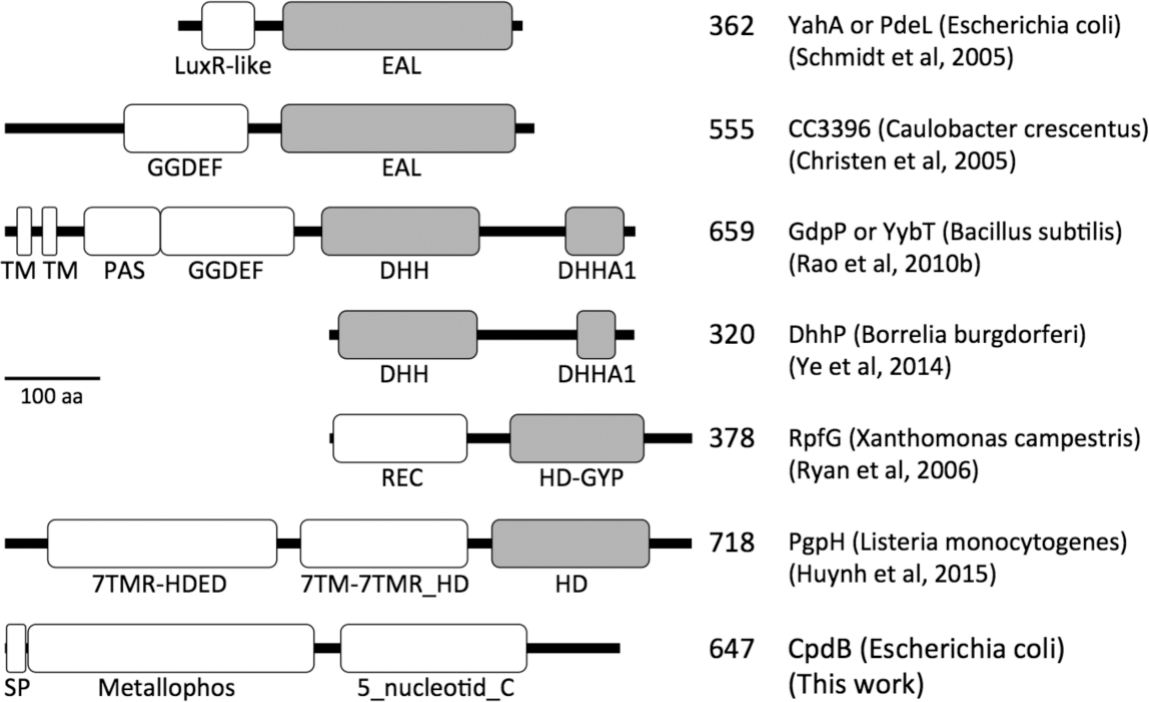
Architectures of cyclic dinucleotide phosphodiesterases of different classes. For comparison with CpdB, six proteins representative of previously known cyclic dinucleotide phosphodiesterases are shown with their catalytic domains highlighted (grey): two EAL domain proteins of different architecture, a membrane-bound and a soluble DHH–DHHA1 proteins, a HD-GYP protein, and a 7TMR-HD protein. CpdB does not contain any of those domains. TM, transmembrane helix. SP, cleavable signal peptide addressing the precursor protein to the periplasm; not present in the mature protein. The numbers indicate the length of the proteins.

Known enzymes that hydrolyze bacterial cyclic dinucleotides are phosphodiesterases that form linear dinucleotides as products, which in some cases are also hydrolyzed to 5´-nucleotides. They belong to four different (sub)classes characterized by different protein domains or combinations (Fig 7) and with different substrate preferences. The phosphodiesterases with preference for c-di-GMP contain either an EAL domain [51–58] or a HD domain with a GYP motif (HD-GYP) [55, 59–64], while phosphodiesterases with preference for c-di-AMP contain either a HD domain bound to a 7TM receptor (7TMR-HD proteins) [65, 66] or both DHH and DHHA1 domains (DHH–DHHA1 proteins) [32, 34, 67–70]. CpdB is structurally unrelated to those cyclic dinucleotide phosphodiesterases. The analysis of CpdB sequence against the Conserved Domains database showed that it does not contain EAL, HD, or DHH domains (Fig 7). Instead, CpdB contains two different domains: (i) a N-terminal metallophos domain also known as calcineurin-like phosphoesterase domain (Pfam ID PF00149), typical of the metallophosphoesterases or “metallo-dependent phosphatases” superfamily (SCOP2 ID 3001067); and (ii) a 5´-nucleotidase, C-terminal domain (5_nucleotid_C; PF02872). These domains are structurally related to those of the archetypical 5´-nucleotidase UshA [71]. It must be emphasized that, despite the structural relatedness to UshA, CpdB did not hydrolyze 5´-AMP but 3´-AMP (see above). It is also important to notice that HD (PF01966) and DHH (PF01368) domains are present in proteins named as metal-dependent phosphohydrolases or phosphoesterases which however are members of protein superfamilies unrelated to the metallophosphoesterases superfamily (SCOP2 ID 3001067) to which CpdB and UshA belong [72].

In conclusion, the finding that CpdB hydrolyzed in vitro bacterial cyclic dinucleotides with significant efficiency could add a novel protein class to the group of cyclic dinucleotide phosphodiesterases. This fulfills predictions that the spectrum of phosphodiesterases active on cyclic dinucleotides could expand to additional protein families [70, 73]. In this regard, it is also worthy of mention that in *Streptomyces* species, which are known to synthesize c-di-AMP, no c-di-AMP phosphodiesterase has been identified [74] while, on the other hand, they contain CpdB homologs.

Considering the in vitro behavior of CpdB as a cyclic dinucleotide phosphodiesterase, its specificity resembles DHH–DHHA1 phosphodiesterases in their preference for c-di-AMP over c-di-GMP [32, 34, 67–70]. In the cases of *Streptococcus pneumoniae* Pde2 [67] and *Mycobacterium tuberculosis* CnpB (also known as Rv2837c or MtbPDE) [69], the resemblance includes that, like CpdB, they form 5´-AMP as product without detection of the linear pApA. Most likely this is so because the linear dinucleotide is better substrate than the cyclic dinucleotide (Table 1, Fig 6).

The catalytic efficiency of CpdB acting on c-di-AMP (2.9×10^4^ M^-1^s^-1^) can be compared with the few DHH–DHHA1 cyclic diadenylate phosphodiesterases for which the *k*_cat_/*K*_m_ ratio is available: it is lower than the efficiency of *B*. *subtilis* GdpP or YybT (4.2×10^5^ M^-1^s^-1^) [34], but higher than those of *S*. *pneumoniae* Pde1 (4.0×10^3^ M^-1^s^-1^) and Pde2 (1.8×10^3^ M^-1^s^-1^) [67]. Concerning the EAL and HD-GYP cyclic diguanylate phosphodiesterases, they display catalytic efficiencies between 2×10^1^ M^-1^s^-1^ and 2×10^5^ M^-1^s^-1^ [61], a range within which the efficiency of CpdB on c-di-GMP fits also well. Anyhow, quantitative comparisons of the catalytic efficiencies of CpdB with those of the known cyclic dinucleotide phosphodiesterases would be more reliable if performed strictly in parallel.

The broad specificity of CpdB contrasts with the reported specificity of other cyclic dinucleotide phosphodiesterases. However, there are few extensive studies of the substrate specificity of these enzymes to allow comparison with CpdB. In one detailed study of the specificity of *B*. *subtilis* GdpP, among the substrates hydrolyzed by CpdB (Table 1), GdpP does not hydrolyze 3´-AMP, linear dinucleotides, bis-5´,5´´´-diadenosine oligophosphates or 4-nitrophenylphosphate, but hydrolyzes 2´,3´-cAMP, ATP and bis-4-nitrophenylphosphate [34, 75]. For many other cyclic dinucleotide phosphodiesterases, the description of their specificity is based on the preference for one or another cyclic dinucleotide, for cyclic versus linear dinucleotides, or on the absence of activity over the cyclic mononucleotides 3´,5´-cAMP or 3´,5´-cGMP [51–53, 66, 76, 77]. Although these criteria are valid for the purposes they were used, they were not accompanied by assays of possible activities on the diversity of compounds tested as substrates of CpdB (Table 1) or GdpP [34, 75]. Therefore, for many cyclic dinucleotide phosphodiesterases, it remains to be seen what is their degree of specificity among a wide variety of nucleotidic compounds.

### The action of CpdB on cyclic dinucleotides could be related to the virulence of the *cpdB* gene or to the environmental interaction with bacteria producing cyclic dinucleotides

It has been reported that the *cpdB* gene has effects on bacterial virulence. When first tested in orogastric and intravenous mouse infection models, the *cpdB* mutant of *Y*. *enterocolitica* showed no significant change in virulence as judged by the numbers of bacteria recovered in spleen, liver, Peyer’s patches or mesenteric lymph nodes 4–5 days after infection [4]. However, the possible contribution of *cpdB* to virulence has been more recently re-examined using a mutant of *S*. *enterica* serovar Pullorum in an intraperitoneal chicken infection model [6]. In this system, the course of infection was followed for up to 16 days, based on the known intracellular persistence of S. Pullorum in splenic macrophages and other locations [78, 79]. Comparing the infection course with the wild-type or the *cpdB* mutant of *S*. *enterica*, it was observed that the numbers of bacteria present in the chicken liver, spleen and cecum differed little for up to 5–6 days post infection, but after this time the *cpdB* mutant disappeared rapidly, while the wild type persisted in high numbers for the whole duration of the experiment [6]. According to the enzyme specificity of *E*. *coli* CpdB, and related to its major activities on 3´-AMP and 2´,3´-cyclic mononucleotides, there is no obvious rationale for the effect of *cpdB* on the intracellular persistence of *S*. *enterica*. On the contrary, it is tempting to speculate whether it could be related to the activity of CpdB on cyclic dinucleotides, which are considered as pathogen-associated molecular patterns (PAMP) that trigger innate immunity [80–86].

On a different scenario, one could also speculate whether the occurrence of extracytoplasmic cyclic dinucleotide phosphodiesterases like CpdB in *E*. *coli* could play a role in the environmental interaction with other bacteria. For instance, the liberation of cyclic dinucleotides by the bacterial mass of the human intestine is viewed as a potential and dangerous source of pro-inflammatory effects in the local epithelium [20]. Periplasmic CpdB of commensal bacteria of the gut could act as a protective factor by removing those compounds.
